# Enhanced Effect of IL-1*β*-Activated Adipose-Derived MSCs (ADMSCs) on Repair of Intestinal Ischemia-Reperfusion Injury via COX-2-PGE_2_ Signaling

**DOI:** 10.1155/2020/2803747

**Published:** 2020-04-17

**Authors:** Liu Liu, Yi Ren He, Shao Jun Liu, Lei Hu, Li Chuang Liang, Dong Liang Liu, Lin Liu, Zhi Qiang Zhu

**Affiliations:** ^1^Department of General Surgery, The First Affiliated Hospital of the University of Science and Technology of China, China; ^2^Department of General Surgery, Anhui Provincial Hospital Affiliated to the Anhui Medical University of China, China; ^3^Department of Anaesthesiology, The First Affiliated Hospital of the University of Science and Technology of China, China

## Abstract

Adipose-derived mesenchymal stem cells (ADMSCs) have been used for treating tissue injury, and preactivation enhances their therapeutic effect. This study is aimed at investigating the therapeutic effect of activated ADMSCs by IL-1*β* on the intestinal ischaemia-reperfusion (IR) injury and exploring potential mechanisms. ADMSCs were pretreated with IL-1*β* in vitro, and activation of ADMSCs was assessed by *α*-SMA and COX-2 expressions and secretary function. Activated ADMSCs was transplanted into IR-injured intestine in a mouse model, and therapeutic effect was evaluated. In addition, to explore underlying mechanisms, COX-2 expression was silenced to investigate its role in activated ADMSCs for treatment of intestinal IR injury. When ADMSCs were pretreated with 50 ng/ml IL-1*β* for 24 hr, expressions of *α*-SMA and COX-2 were significantly upregulated, and secretions of PGE_2_, SDF-1, and VEGF were increased. When COX-2 was silenced, the effect of IL-1*β* treatment was abolished. Activated ADMSCs with IL-1*β* significantly suppressed inflammation and apoptosis and enhanced healing of intestinal IR injury in mice, and these effects were impaired by COX-2 silencing. The results of RNA sequencing suggested that compared with the IR injury group activated ADMSCs induced alterations in mRNA expression and suppressed the activation of the NF-*κ*B-P65, MAPK-ERK1/2, and PI3K-AKT pathways induced by intestinal IR injury, whereas silencing COX-2 impaired the suppressive effect of activated ADMSCs on these pathway activations induced by IR injury. These data suggested that IL-1*β* pretreatment enhanced the therapeutic effect of ADMSCs on intestinal IR injury repairing via activating ADMSC COX-2-PGE_2_ signaling axis and via suppressing the NF-*κ*B-P65, MAPK-ERK1/2, and PI3K-AKT pathways in the intestinal IR-injured tissue.

## 1. Introduction

Intestinal ischaemia and reperfusion (IR) is a common clinical problem that remains one of the leading causes of mortality in critically ill patients. Intestinal IR injury is a pathological process for a variety of diseases, like superior mesenteric artery (SMA) embolism, intestinal transplantation, radiation enteropathy, and sepsis [1–3]. It has been reported that mucosal barrier damage is prominent during intestinal IR process and leads to gut dysfunction, such as bacterial translocation and decreased nutrient absorption. Most importantly, intestinal IR causes the release of multiple proinflammatory cytokines [4], leading to multiple organ failure and death [5]. Therefore, ameliorating intestinal IR injury and enhancing the repair of IR injury are critical for reducing mortality in patients with critical illness.

Adipose-derived mesenchymal stem cells (ADMSCs) can be differentiated into multiple stromal lineages, including cardiac myocytes and bone and muscle cells [6]. ADMSCs represent a promising avenue for the treatment of a variety of inflammatory and autoimmune diseases, including cardiac failure, spinal cord injury, sepsis, and intestinal IR injury [7–11]. On the one hand, ADMSCs can be differentiated into bone cells for repairing bone fracture [12]; on the other hand, ADMSCs secrete a number of cytokines to enhance tissue regeneration, suppress inflammatory reactions, and alleviate the severity of sepsis and organ injury [7, 13]. Our previous study has proved that ADMSCs were efficacious for enhancing the healing of gastric perforation by promoting tissue regeneration and suppressing inflammation [14]. These evidences suggested that ADMSCs have the potential to treat intestinal IR injury characterized by excessive cellular apoptosis and inflammation.

Notably, it has been reported that interleukin-1*β* (IL-1*β*) pretreatment could enhance the immunomodulatory effect of ADMSCs for treatment of tissue injury characterized by a predominantly inflammatory reaction [15–18]. A previous study confirmed that IL-1*β* significantly enhanced the efficacy of MSCs to attenuate the development and severity of dextran sodium sulphate- (DSS-) induced colitis and that these effects were partially mediated by increased immunosuppressive capacity and enhanced migratory ability [15]. Luo et al. reported that pretreatment with IL-1*β* and transforming growth factor-*β* (TGF-*β*) induced P38 MAPK and SMAD3 activation in MSCs, which significantly improved vascular endothelial growth factor (VEGF) secretion and attenuated myocardial injury caused by acute ischaemia [16]. These findings suggested that pretreatment with inflammatory factors, such as IL-1*β*, might be an effective approach to enhance the efficacy of ADMSCs in the treatment of intestinal IR injury.

In the present study, we demonstrated that IL-1*β* pretreatment activated ADMSCs, reflected by enhanced paracrine function of ADMSCs and activation of COX-2-PGE_2_ signaling axis. IL-1*β*-activated ADMSCs significantly enhanced the repairing effect of intestinal IR injury in a mouse model through decreasing cellular apoptosis and suppressing inflammation. Regarding mechanisms, activated ADMSCs regulated the expression of a number of genes related to “neutrophil chemotaxis,” “inflammatory response,” “chemotaxis,” and “defence response” in vivo and inhibited the activation of the NF-*κ*B-P65, MAPK-ERK1/2, and PI3K-AKT pathways in the IR-injured intestine.

## 2. Materials and Methods

### 2.1. Cell Culture and Treatment

Human ADMSCs were purchased from Cyagen Biosciences (Soochow, China). The ADMSCs were obtained from C57BL/6 mice and isolated, cultured, and expanded according to a previously reported protocol [19]. The cells were maintained in DMEM (Gibco, USA) supplemented with 10% foetal bovine serum, 100 units/ml penicillin, and 100 mg/ml streptomycin. ADMSCs isolated from mice were used at passages 2-10 for subsequent experiments. All animal experimental procedures were approved by the laboratory ethical committee of Anhui Medical University of China (LLSC20150028).

### 2.2. Flow Cytometry and Differentiation

ADMSC phenotypes were identified using flow cytometry, and the cell surface markers CD29 (ab21845), CD90 (ab124527), and CD45 (ab197730) were identified according to a previously reported protocol [14]. Adipogenic and osteogenic differentiation was induced in ADMSCs using differentiation kits (Invitrogen, Life Technologies) according to the manufacturer's instructions.

### 2.3. Establishment of Stable Cells

A lentiviral short-hairpin RNA vector for COX-2 (shCOX-2) and a control vector (shNC) were designed and purchased from GenePharma (Suzhou, China). To generate a lentiviral virus, the shRNA vectors were cotransfected into HEK293T cells along with envelope (VSVG) and packing (Delta 8.9) plasmids as previously described [20]. ADMSCs were infected in the presence of 8 *μ*g/ml polybrene. Following infection for 48 hours, the cells were selected with 2.0 *μ*g/ml puromycin (Sigma). Knockdown efficiencies were confirmed via real-time PCR and western blot analysis.

### 2.4. Cell Treatment

After the cultures reached 80% confluence, ADMSC, ADMSCs/shNC, or ADMSCs/COX-2 cells were pretreated with IL-1*β* (SRP3083, Sigma) at the indicated concentrations and time points. To investigate the role of the COX-2-PGE_2_ signaling axis, ADMSCs were pretreated with IL-1*β*, 50 *μ*M NS398 (a COX-2-specific inhibitor, S8433, Selleck), 5 *μ*M PGE_2_ (P0409, Sigma), or a combination of these factors, as indicated.

### 2.5. Cell Proliferation

A total of 2 × 10^3^ ADMSC cells were treated with or without IL-1*β*, and proliferation of these cells were measured using CCK8 kit according to the manufacturer's protocol.

### 2.6. Migration

ADMSC migration ability was assessed using 0.8 *μ*m Transwell system. In brief, a total of 1 × 10^3^ ADMSC cells treated with or without IL-1*β* were seeded into the upper chamber and cultured for 24 hrs; migrated cells were stained with crystal violet.

### 2.7. Western Blot Analysis

Total protein was extracted from cells or intestinal tissue using a RIPA buffer containing a protease inhibitor cocktail (Sigma). Equal amounts of total protein (20 *μ*g) were separated by SDS-PAGE and transferred to PVDF membranes. After incubation with 5% nonfat milk for 30 min, the membranes were incubated with the following primary antibodies at 4°C for 24 hr: anti-COX-2 (ab15191, Abcam), anti-p21 (#10355-1-AP, Proteintech Group), anti-p25(18742-1-AP, Proteintech Group), anti-phospho-P65 (#3033, Cell Signaling Technology, USA), anti-P65 (#8242, Cell Signaling Technology, USA), anti-phospho-ERK1/2 (#4370, Cell Signaling Technology, USA), anti-ERK1/2 (#4695, Cell Signaling Technology, USA), anti-phospho-AKT (#9275, Cell Signaling Technology, USA), and anti-AKT (#9272, Cell Signaling Technology, USA). The membranes were incubated with *β*-actin antibodies (#5125, Cell Signaling Technology, USA) as an internal control, and the protein bands were visualized using an ECL kit.

### 2.8. Immunofluorescence Staining

Immunofluorescence staining was performed according to a previously published protocol using the following primary antibodies: anti-COX-2 (ab15191, Abcam) and anti-*α*-SMA (ab5694, Abcam).

### 2.9. ELISA Assay

The concentrations of PGE_2_ (ab133021, Abcam), SDF-1 (ab100637, Abcam), VEGF (ab100663, Abcam), IL-10 (ab100549, Abcam), and TGF-*β*1 (ab100647, Abcam) in culture media were measured using commercial ELISA kits according to the manufacturer's protocols.

### 2.10. Total RNA Extraction and Real-Time RT-PCR

Total RNA was isolated from the mouse intestinal tissue using a total RNA purification kit (QIAGEN) according to the manufacturer's protocol. cDNA was synthesized by reverse transcription using a cDNA synthesis kit (Takara, Japan). Real-time polymerase chain reactions (PCR) were performed using a PrimeScript® RT reagent kit (Takara, Japan) on an Applied Biosystems 7500 Real-time PCR system. Data were analysed with the 2-^*ΔΔ*Ct^ method, and *β*-actin was used as an internal control. The primers used for real-time RT-PCR are listed in Supplementary Table 1.

### 2.11. Animal Model

This study was conducted according to the guidelines for the Care and Use of Laboratory Animals of Anhui Medical University. The intestinal IR injury model was produced according to a modified protocol [4]. Briefly, C57BL/6 mice weighing between 20 and 25 g were fasted with free access to water for 12 hr before the experiment. Anaesthesia was conducted via an intraperitoneal injection of 1.5% pentobarbital sodium (100 *μ*l in each mouse). After the skin was disinfected with 1% iodophor, a vertical abdominal incision was made. The SMA was identified and clipped for 1 h. Just after the clamp was released, cells (ADMSCs, IL-1*β*-pretreated ADMSCs, IL-1*β*-pretreated ADMSCs/shNC, or IL-1*β*-pretreated ADMSCs/shCOX-2) suspended in 600 *μ*l of phosphate-buffered saline (PBS) were locally injected into the submucosa of the ischaemic intestinal segment at 10 different sites. In the sham group, mice underwent laparotomy without intestinal IR injury, and in the IR group, 600 *μ*l of PBS was locally injected instead of cells.

Two sets of animal experiments were conducted. First, to explore whether IL-1*β*-pretreated ADMSCs accelerated intestinal IR injury healing, the following four groups of C57BL/6 mice were used: (1) a sham group, (2) an IR group, (3) an ADMSC group in which IR-treated mice received a total of 5 × 10^6^ ADMSCs, and (4) an IL-1*β*-pretreated ADMSC group in which IR-treated mice received 5 × 10^6^ IL-1*β*-pretreated ADMSCs (ADMSCs were stimulated with 50 ng/ml IL-1*β* for 24 hr before transplantation). Second, to explore whether COX-2 played a critical role in the effects of IL-1*β*-pretreated ADMSCs in the treatment of intestinal IR injury, the following two groups of mice were used: (5) an IL-1*β*-pretreated ADMSC/shNC group in which 5 × 10^6^ IL-1*β*-pretreated ADMSCs/shNC were used and (6) an IL-1*β*-pretreated ADMSCs/shCOX-2 group in which 5 × 10^6^ IL-1*β*-pretreated ADMSCs/shCOX-2 were used. The abdominal wall was closed, and the mice were allowed to recover with standard care and monitoring. Six mice were used per group.

Mice were humanely killed at 2 days after surgery, and intestinal segments were dissected and stored in 4% paraformaldehyde for histological analysis and at -80°C for western blot analysis and RNA extraction, respectively.

### 2.12. Histological Analysis

Paraffin-embedded intestinal tissue was cut into 4 *μ*m-thick slides for haematoxylin and eosin staining. Five fields in each sample were randomly chosen for pathological analysis by two independent pathologists who were blinded to the treatments. The histological scale reported by Chiu et al. was used to assess the severity of intestinal IR injury [21].

### 2.13. Myeloperoxidase (MPO) Activity

MPO activity was measured using a Myeloperoxidase Activity Assay Kit (ab105136, Abcam) according to the manufacturer's instructions.

### 2.14. Apoptosis

Apoptotic cells were detected using a TUNEL assay kit (ab66110, Abcam) according to the manufacturer's protocol. Five fields (at 200× manification) in each sample were randomly selected and quantified using ImageJ software (National Institutes of Health, USA).

### 2.15. mRNA Sequencing

In brief, total RNA was extracted from IL-1*β*-pretreated ADMSCs and the IR group (*n* = 2 mice for each group) using a TRIzol reagent (Invitrogen, CA, USA) according to the manufacturer's protocol. The quantity and purity of the total RNA were analysed using a Bioanalyzer 2100 and RNA 6000 Nano LabChip Kit (Agilent, CA, USA) with RIN number > 7.0, respectively. Approximately 10 *μ*g of total RNA was used to isolate poly (A)-mRNA with poly-T oligo-attached magnetic beads (Invitrogen, USA). Following purification, the mRNA was fragmented into small pieces at an elevated temperature. Then, the cleaved RNA fragments were reverse transcribed to create the final cDNA library in accordance with the protocol included in the mRNA-Seq sample preparation kit (Illumina, San Diego, USA). Then, we performed paired-end sequencing on an Illumina Hiseq (4000 LC Sciences, USA) according to the vendor's recommended protocol. The mapped reads obtained in each sample were assembled using StringTie. Then, all transcriptomes obtained from the samples were merged to reconstruct a comprehensive transcriptome using Perl scripts. After the final transcriptome was generated, StringTie and Ballgown were used to estimate the expression levels of all transcripts. StringTie was used to determine the expression levels of mRNAs by calculating the fragments per kilobase of the exon model per million mapped reads (FPKM). The differentially expressed mRNAs were selected as log2 (fold change, FC) > 1 or log2 (FC)<−1 and with a *P* value < 0.05 in the Ballgown R package. The mRNA expression sequencing and data processing were performed by Lian Chuan Biotech, Hangzhou, China.

### 2.16. GO Enrichment and KEGG Pathway Analyses

Gene enrichment ontology (GO) analysis (http://www.geneontology.org) was used to explore the functions of differentially expressed mRNAs between the two groups, and the different functions were classified into three categories: biological processes, cellular components, and molecular functions. A *P* value < 0.05 indicated significant GO term enrichment in the deregulated expressed genes.

The pathway analysis focused on differentially expressed mRNAs in the “biological process” category of the GO analysis and was performed according to the Kyoto Encyclopedia of Genes and Genomes (KEGG, https://www.genome.jp/kegg). This was because deregulated mRNAs in the “biological process” category are more important for IR injury treatment with IL-1*β*-pretreated ADMSCs. A *P* value < 0.05 suggested a significant role for the pathway in intestinal IR injury treated with IL-1*β*-pretreated ADMSCs.

### 2.17. Statistical Analysis

All data are presented as the mean ± standard deviation (SD), and each experiment was repeated at least three times. Student's *t* test was used for comparisons between two groups, and one-way ANOVA with Bonferroni's test was performed for multiple group comparisons. All statistical tests were two tailed, and *P* < 0.05 indicated statistical significance. All statistical analyses were conducted using IBM SPSS v. 22.

## 3. Results

### 3.1. Differentiation and Characteristics of ADMSCs

ADMSCs were spindle shaped and induced to differentiate into adipocytes and osteoblasts (Supplementary Fig. A-C). Additionally, flow cytometric analysis demonstrated that ADMSCs expressed high levels of CD29 and CD90 and low levels of CD45, in line with our previous study (Supplementary Fig. 1D-F).

### 3.2. IL-1*β* Activated ADMSCs through COX-2-PGE_2_ Signaling

**Figure 1:**
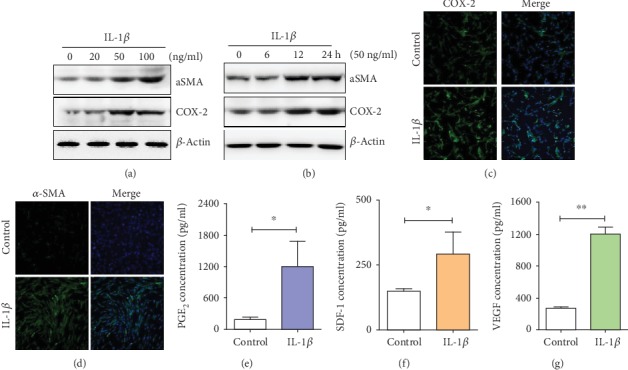
The effect of IL-1*β* pretreatment on ADMSC activation. (a) Expressions of *α*-SMA and COX-2 were upregulated at a dose-dependent manner when ADMSCs were treated with different concentrations of IL-1*β* (0, 20, 50, and 100 ng/ml) for 12 hr. (b) Expressions of *α*-SMA and COX-2 were upregulated at a time-dependent manner when ADMSCs were treated with 50 ng/ml IL-1*β* for different times (0, 6, 12, and 24 hrs). (c, d) Immunofluorescence staining indicated that IL-1*β* (50 ng/ml) treatment increased the expression of *α*-SMA and COX-2 in ADMSCs. (e–g) IL-1*β* treatment increased the secretion of PGE_2_, VEGF, and SDF-1 (^∗^*P* < 0.05 and ^∗∗^*P* < 0.01, Student's *t* test).

Expression levels of *α*-SMA and COX-2, biological markers of activated ADMSC, were upregulated when human ADMSCs were treated with different concentrations of IL-1*β* (0, 20, 50, or 100 ng/ml) for 12 hr. Importantly, 50 ng/ml IL-1*β* induced the COX-2 expression at most, and this concentration was therefore selected for further experiments (Figure 1(a)). Human ADMSCs were then treated with 50 ng/ml IL-1*β* for different times (0, 6, 12, and 24 hr), and expression levels of *α*-SMA and COX-2 were upregulated in a time-dependent manner (Figure 1(b)); immunofluorescence staining confirmed that *α*-SMA and COX-2 expression was increased with 50 ng/ml IL-1*β* treatment for 24 hr (Figures 1(c) and 1(d)). The effect of IL-1*β* was further confirmed in mouse ADMSCs (Supplementary Fig. 2A-B). In addition, to demonstrate whether IL-1*β* treatment enhanced ADMSC secretary function, factors of PGE_2_, SDF-1, VEGF, IL-10, and TGF-*β*1 were measured. More PGE_2_, SDF-1, and VEGF were secreted from ADMSCs treated with 50 ng/ml IL-1*β* for 24 hr (Figures 1(e)–1(g) and Supplementary Fig. 3A-D). It has been reported that inflammation is a determining cause of cellular senescence and aging of ADMSCs, and the senescent ADMSCs furthermore secrete proteins called senescence-associated secretory phenotype (SASP) proteins to carry out several functions, such as immunomodulation and promoting tissue development [22, 23]. We found that IL-1*β*-activated ADMSCs exhibited decreased proliferation and migration when comparing to ADMSCs. In addition, P21 and P25, biological markers involved in cellular senescence, were upregulated in IL-1*β*-activated ADMSCs (Supplementary Fig. 3E-G). Collectively, these results suggested that IL-1*β* pretreatment could induce SASP and effectively activate ADMSCs in vitro.

### 3.3. Inhibiting COX-2 Expression Suppressed the Effect of IL-1*β* on ADMSC Activation

**Figure 2:**
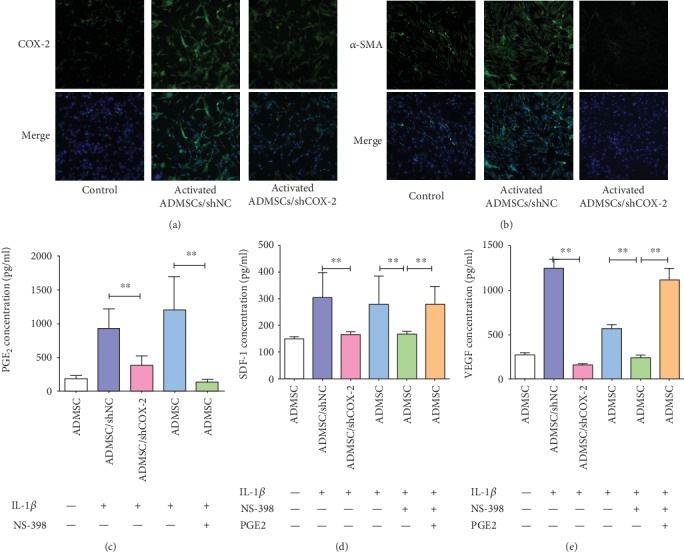
IL-1*β* treatment activated ADMSCs dependent on COX-2-PGE_2_ signaling. (a, b) COX-2 silencing impaired the effect of IL-1*β* on the upregulation of COX-2 and *α*-SMA expressions in ADMSCs. (c) COX-2 silencing impaired enhanced effect of IL-1*β* on PGE_2_ secretion in ADMSCs. (d, e) Silencing and inhibition of COX-2 expression impaired the promoted effect of IL-1*β* on SDF-1 and VEGF secretion in ADMSCs, whereas exogenous PGE_2_ rescued SDF-1 and VEGF secretion when IL-1*β*-pretreated ADMSCs were treated with COX-2 inhibitor, NS398 (^∗∗^*P* < 0.01, Bonferroni's multiple comparison).

COX-2 expression was specifically inhibited by shCOX-2 or NS-398 (a COX-2-specific inhibitor) in ADMSCs with applied alone or in cells subsequently stimulated with IL-1*β* (Figure 2(a), Supplementary 4A-C). COX-2 silencing decreased *α*-SMA expression (Figure 2(b)) and decreased secretion of PGE_2_, SDF-1, and VEGF in ADMSCs treated with IL-1*β* (Figures 2(c)–2(e), Supplementary 3D). Interestingly, exogenous PGE_2_ rescued SDF-1 and VEGF secretion when IL-1*β*-pretreated ADMSCs were treated with NS398 (Figures 2(d) and 2(e)). These findings suggested that IL-1*β* induced ADMSC activation dependent on the COX-2-PGE_2_ signaling in vitro.

### 3.4. Activated ADMSCs Enhanced Healing of Intestinal IR Injury through COX-2-PGE_2_ Signaling In Vivo

**Figure 3:**
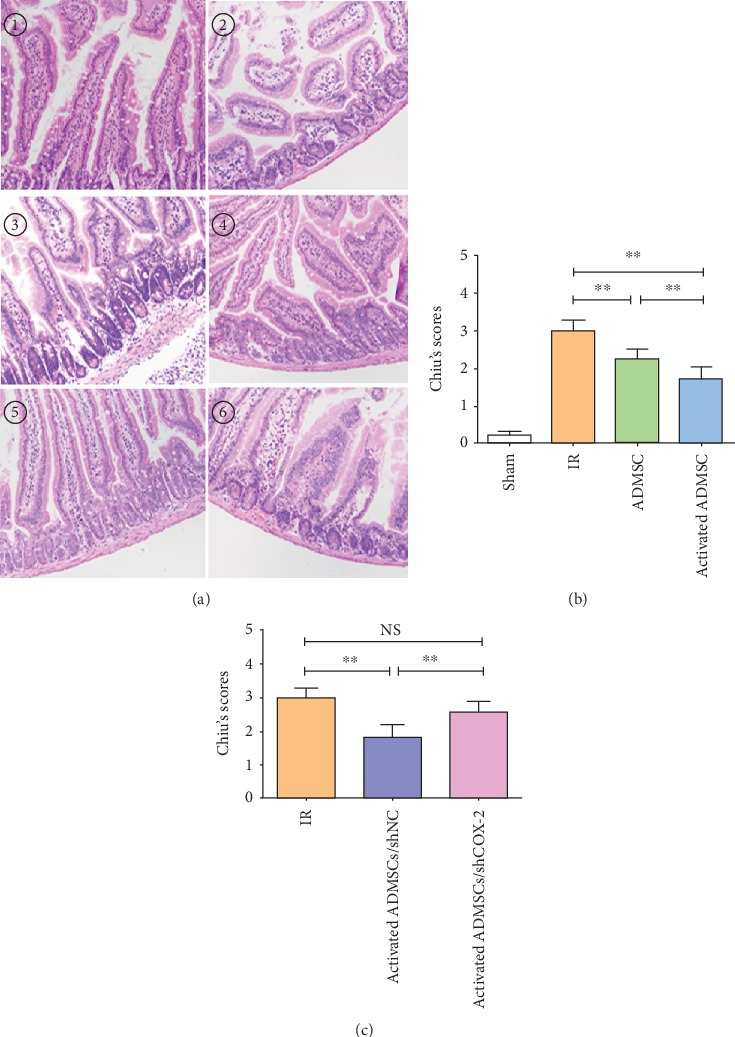
Activated ADMSC enhanced healing of intestinal IR injury dependent on COX-2-PGE_2_ signaling. (a) Representative hematoxylin and eosin staining (HE) pictures for different groups (① sham, ② IR, ③ ADMSCs, ④ activated ADMSC, and ⑤activated ADMSC/shNC or ⑥ activated ADMSC/shCOX-2). (b, c) Chiu's scores for assessment of intestinal IR injury after different treatments (^∗∗^*P* < 0.01, NS: no statistical significance, Bonferroni's multiple comparison).

An intestinal IR injury model was produced in mice, in which ADMSCs or activated ADMSCs with IL-1*β* pretreatment were locally injected. Histological examination showed that intestinal villi were destroyed and massive inflammatory cells were observed in the lamina propria in the IR group. In contrast, the ADMSC group showed less intestinal damage, as evidenced by slight villus destruction and lower levels of inflammatory cell infiltration. Interestingly, activated ADMSCs further enhanced the damage repairing after intestinal IR injury. When COX-2 expression was silenced, the effect of activated ADMSCs was impaired (Figure 3(a)). Chiu's scores were used to assess IR injury, as shown in Figures 3(b) and 3(c).

### 3.5. Apoptosis and Inflammation

**Figure 4:**
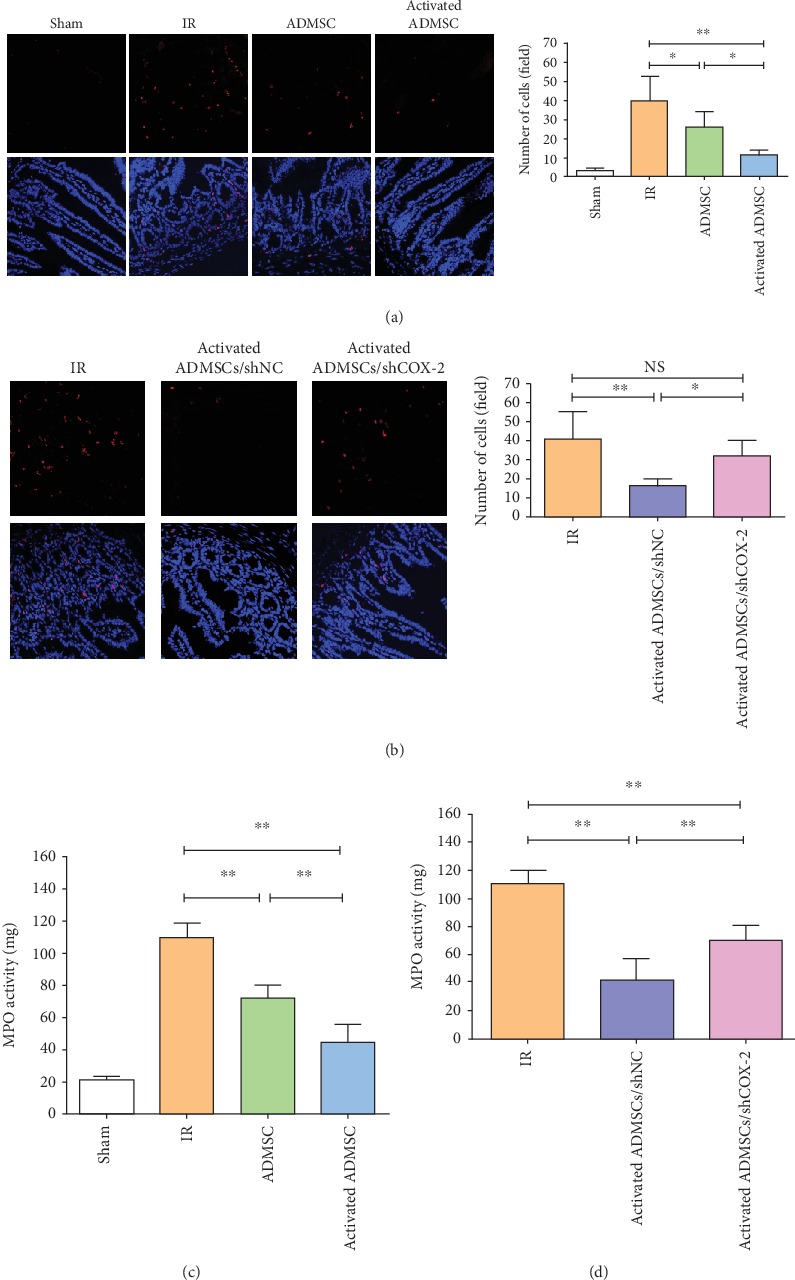
Activated ADMSC decreased apoptosis and neutrophil infiltration in IR-injured intestinal tissue. (a, b) Apoptosis was evaluated by TUNEL staining for different groups. (c, d) Neutrophil infiltration was assessed by MPO activity (^∗^*P* < 0.05, ^∗∗^*P* < 0.01, NS: no statistical significance, Bonferroni's multiple comparison).

Compared with the IR group, ADMSC treatment decreased apoptosis of epithelial cells, and the therapeutic effect of ADMSCs was enhanced by IL-1*β* activation. When COX-2 expression was silenced, the effect of activated ADMSCs was impaired (Figures 4(a) and 4(b)).

**Figure 5:**
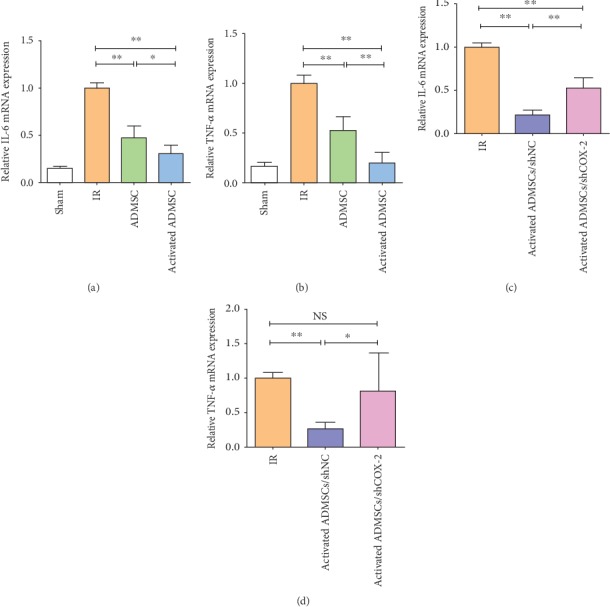
Activated ADMSC decreased inflammation in IR-injured intestinal tissue. (a, c) Expression of IL-6 in different groups. (b, d) Expression of TNF-*α* in different groups (^∗^*P* < 0.05, ^∗∗^*P* < 0.01, NS: no statistical significance, Bonferroni's multiple comparison).

MPO activity was measured to assess neutrophil infiltration among these groups. The results showed that compared with the ADMSC and IR groups activated ADMSCs showed the lowest level of intestinal inflammation and that suppression of COX-2 expression impaired the ability of activated ADMSCs to alleviate intestinal inflammation (Figures 4(c) and 4(d)). Furthermore, the expressions of the inflammatory factors IL-6 and TNF-*α* were detected, and, as expected, the activated ADMSC group had the lowest levels of IL-6 and TNF-*α* (Figures 5(a) and 5(b)) and effect that was eliminated by COX-2 suppression (Figures 5(c) and 5(d)). These data suggested that activation with IL-1*β* improved the therapeutic efficacy of ADMSCs for intestinal IR injury and that COX-2 played a critical role for activated ADMSCs to treat IR injury.

### 3.6. Differential Expressions of mRNAs

**Figure 6:**
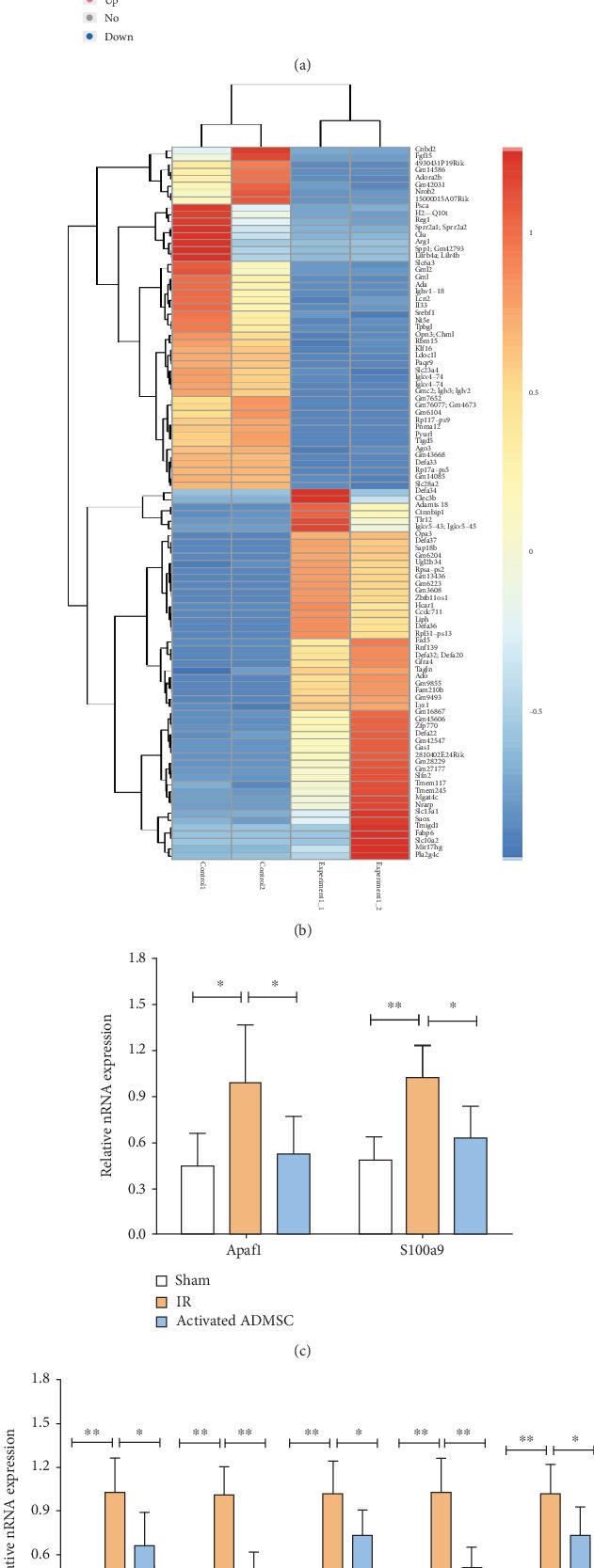
mRNA sequencing revealed that activated ADMSC treatment induced alteration of mRNA expression in comparison with the IR group. (a) Volcano plot showed differentially expressed mRNAs between the activated ADMSC and IR groups. (b) Hierarchical clustering showed the top 100 deregulated mRNAs between the activated ADMSC and IR groups. (c, d) Results of real-time RT-PCR confirmed that seven mRNAs were upregulated in the IR group and down-regulated in activated ADMSC group, which was in lined with results from mRNA sequencing (^∗^*P* < 0.05, ^∗∗^*P* < 0.01, Bonferroni's multiple comparison).

mRNA sequencing was used to explore the differential expression of intestinal mRNAs between the IR and activated ADMSC groups. Compared with the IR group, a total of 907 mRNAs were deregulated (407 mRNAs were upregulated and 500 mRNAs were downregulated) in the activated ADMSC group (Figure 6(a)). Hierarchical clustering showed the top 100 deregulated mRNAs between the activated ADMSC and IR groups (Figure 6(b)).

To validate the results obtained from mRNA sequencing, two apoptosis-related (Apaf1 and S100a9) and five immune response-related (CXCL11, CXCL2, Tnfrsf22, CXCR2, and CXCR4) mRNAs were selected for further detection by real-time RT-PCR. The results showed that all seven mRNAs were expressed at lower levels in the activated ADMSC group than in the IR group, in line with the results obtained from mRNA sequencing (Figures 6(c) and 6(d)).

### 3.7. GO Enrichment Analysis for Biological Functions

**Figure 7:**
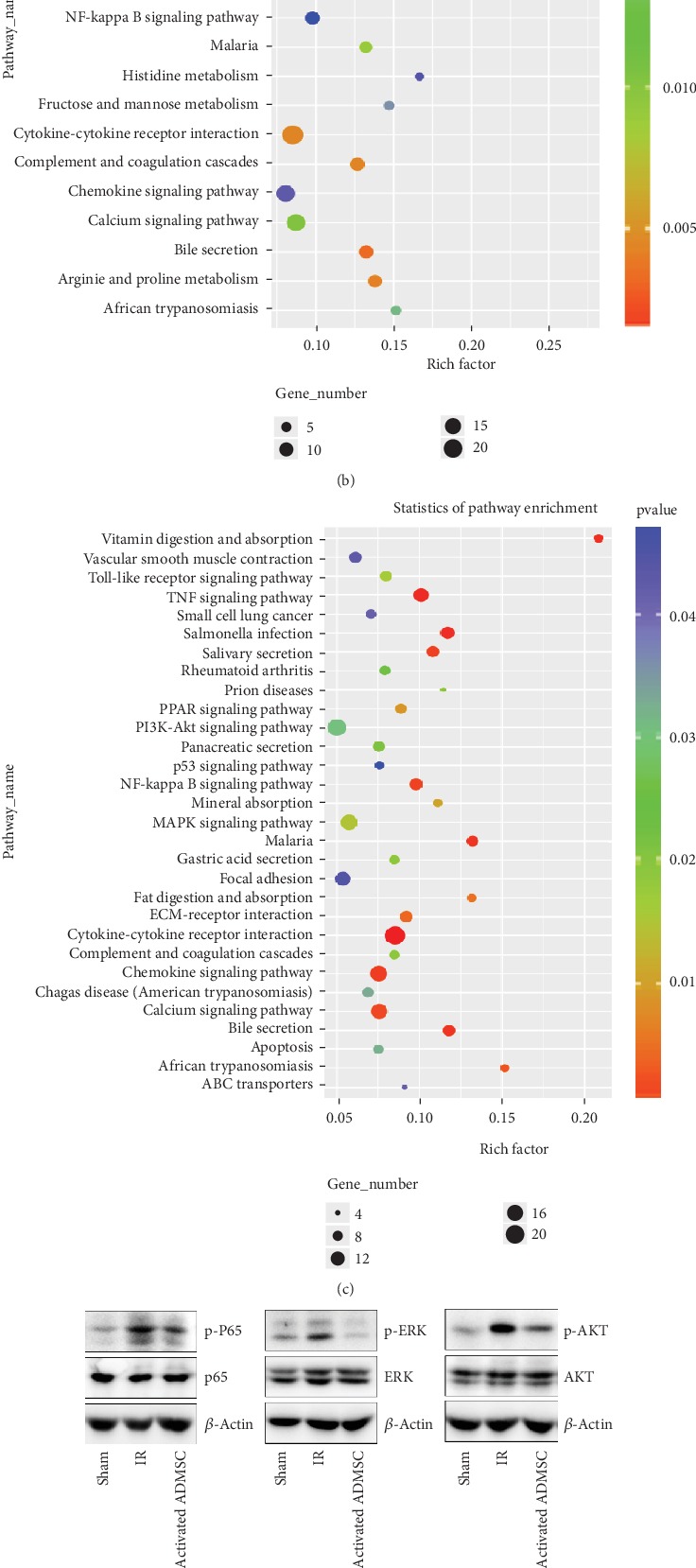
GO enrichment analysis and KEGG pathway analysis were used to explore the possible mechanism of activated ADMSC for treatment of intestinal IR injury. (a) GO enrichment analysis revealed that differentially expressed mRNAs were involved in “biological process,” “cellular component,” and “molecular function” processes. (b) Deregulated mRNAs participated in multiple biological functions like “neutrophil chemotaxis,”, “inflammatory response”, “chemotaxis” and “defense response”. (c) KEGG pathway analysis showed that several pathways (NF-*κ*B-P65, MAPK-ERK, and PI3K-AKT pathways) were involved in activated ADMSC for treatment of intestinal IR injury. (d) Results from western blot showed that activated ADMSC inhibited phosphorylation of P65, ERK, and AKT that were induced by IR injury. (e, f) Silencing of COX-2 impaired the effect of activated ADMSC on inhibition of the seven mRNA expressions and inaction of NF-*κ*B-P65, MAPK-ERK, and PI3K-AKT pathways in intestinal IR-injured tissue.

GO enrichment analysis was conducted to explore mechanisms for activated ADMSCs to enhance the healing of intestinal IR injury. Differentially expressed mRNAs were categorized as “biological process,” “cellular component,” or “molecular function” (Figure 7(a)), and deregulated mRNAs were found to be involved in multiple biological functions, such as “neutrophil chemotaxis,” “inflammatory response,” “chemotaxis,” and “defence response” (Figure 7(b)), all of which are closely related to the repair process in the intestinal IR injury.

### 3.8. KEGG Pathway Analysis

Because “biological process” (including “inflammatory response,” “neutrophil chemotaxis,” and “chemotaxis”) was closely related to healing process of IR injury treated with activated ADMSCs, KEGG pathway analysis was conducted based on the deregulated mRNAs involved in “biological process in GO enrichment analysis”. A total of 253 mRNAs were upregulated, while 263 mRNAs were downregulated, and KEGG pathway analysis showed that these mRNAs are involved in several signal pathways, including the “TNF signaling pathway”, “MAPK signaling pathway”, “PI3K-Akt signaling pathway”, and “NF-*κ*B signaling pathway” (Figure 7(c)). As demonstrated by western blot analyses, the expression levels of phospho-P65, phospho-ERK, and phospho-AKT were significantly higher in the IR group than in the sham group and were remarkably decreased in activated ADMSC group (Figure 7(d)).

### 3.9. Inhibiting COX-2 Expression in Activated ADMSCs on Apoptosis-Immune-Related Gene Expression and Signaling Pathways

As shown in Figures 6(c) and 6(d), seven mRNAs (apoptosis-related genes: Apaf1 and S100a9; immune-related genes: CXCL11, CXCL2, Tnfrsf22, CXCR2, and CXCR4) were expressed at lower level in the activated ADMSC group than in the IR group. Considering that COX-2 is critical for the biological functions of activated ADMSCs, it was worth investigating the expression of these mRNAs when COX-2 was silenced. As expected, these mRNAs were expressed at significantly lower levels in the activated ADMSC group compared with the IR group; however, when COX-2 expression was suppressed, the effect of activated ADMSCs was impaired (Figures 7(f) and 7(g)).

As shown above, activated ADMSCs repressed the NF-*κ*B-P65, MAPK-ERK1/2, and PI3K-AKT pathways in IR-injured intestines. We then further evaluated the influence of COX-2 suppression on these signaling pathways. As demonstrated by western blot, the levels of phosphorylation of P65, ERK1/2, and AKT were significantly lower in activated ADMSCs than in the IR group, and COX-2 silencing abolished the effect of activated ADMSCs on these pathways in intestinal IR-injured tissues (Figure 7(e)).

## 4. Discussion

**Figure 8:**
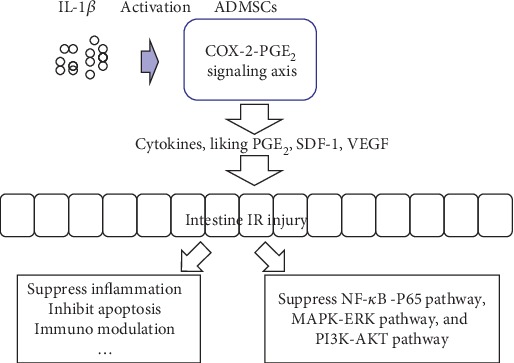
Activated ADMSC enhanced healing of intestinal IR injury through activation of COX-2-PGE_2_ signal axis and transplanted activated ADMSC-suppressed apoptosis and inflammation and inhibited activation of NF-*κ*B-P65, MAPK-ERK, and PI3K-AKT pathways that were induced by IR injury.

MSCs have received a growing attention due to their therapeutic effects in many serious diseases, such as intestinal IR injury, that could not be effectively treated till now. Because of its easy availability, fat tissue has been recognized as an important source of MSCs, as is the bone marrow. Many studies have emphasized the importance of the activation of MSCs in an inflammatory environment for the treatment of organ injury [18, 24–26]. Gonzalez-Rey et al. reported that the protective effect of MSCs was not observed in DSS colitis due to the absence of inflammatory cytokine release, which was proposed to induce MSC activation, when MSCs were injected into the normal gut before model induction [27]. However, MSCs enhanced the healing of DSS-induced colitis when the cells were administered into the inflammatory bowel [27, 28]. Another study reported similar results which were that IFN-*γ*-activated MSCs but not nonactivated MSCs were efficacious for treatment of DSS-induced colitis [29]. These data indicated that the activation of MSCs by inflammatory factors in vitro or in vivo is very important. In the present study, we evaluated the effect of IL-1*β* on ADMSC activation in vitro and the therapeutic efficacy of IL-1*β*-activated ADMSCs on intestinal IR injury in vivo. In addition, we also focused on the role of COX-2-PGE_2_ signaling axis in IL-1*β*-induced ADMSC activation, because COX-2-PGE_2_ signaling can effectively convert the inflammatory environment to an anti-inflammatory status by imposing a significant immunomodulatory effect on both macrophages and T cells [30, 31], a process that is critical for the healing of intestinal IR injury. The results of our study demonstrated that COX-2-PGE_2_ signaling was critical for IL-1*β* to activate ADMSCs. When COX-2 expression was inhibited, the effect of IL-1*β* on ADMSC activation was abolished, and the therapeutic effect of activated ADMSCs was impaired in intestinal IR injury (Figure 8).

Some important findings were presented in our study. First, we demonstrated potential mechanisms for IL-1*β* to activate ADMSCs, which has not been fully investigated in previous studies. Our results showed that by activating COX-2-PGE_2_ signaling, IL-1*β* treatment significantly induced ADMSCs to secrete an amount of PGE_2_, VEGF, and SDF-1. However, suppression of COX-2 expression abrogated the effect of IL-1*β* on ADMSC activation, which could be rescued by exogenous PGE_2_ treatment. This mechanism has also been reported for other inflammatory factors, including TNF-*α* and interleukin-17A [24, 26], suggesting that COX-2-PGE_2_ signaling was pivotal for IL-1*β*-induced ADMSC activation.

The second important finding of our study was that we evaluated the therapeutic effects of ADMSCs (including ADMSCs, activated ADMSCs, and activated ADMSCs/shCOX-2) on the healing of intestinal IR injury in mice. So far, no study evaluated the therapeutic effect of activated ADMSCs with IL-1*β* for repairing intestinal IR injury, and the underlying mechanisms were not fully understood. In the present study, activated ADMSCs were the most effective for the treatment of IR injury, reflected by the lowest severity of IR injury and the lowest levels of apoptosis and inflammation, whereas activated ADMSCs/shCOX-2 displayed the worst efficacy. Li et al. reported that MSCs significantly protected rats against hepatic IR injury and were associated with lower serum levels of liver enzymes and reduced neutrophil infiltration and expression of apoptosis-related genes [32]. Another study reported that pretreatment with TNF-*α*, IL-1*β*, and nitric oxide enhanced the paracrine functions of MSCs and that conditioned medium obtained from pretreated MSCs reduced the inflammatory reaction to radiation-induced intestinal injury and enhanced epithelial cell proliferation [33]. A study conducted by Bai et al. further demonstrated that IL-17A-pretreated MSCs ameliorated IR-induced acute kidney injury, reduced renal inflammation, and increased the percentage of Tregs in the kidneys, while using celecoxib to block COX-2 reversed the benefits of IL-17A-pretreated MSCs, suggesting a pivotal role for COX-2 [24]. We found that activated ADMSCs significantly reduced MPO activity, inflammation, and epithelial apoptosis, as indicated by the downregulated expressions of IL-6 and TNF-*α* and TUNEL staining in IR-injured intestines. As expected, the benefits of activated ADMSCs were impaired when COX-2 expression was inhibited. Collectively, our results, along with those presented in previous studies, suggested that pretreatment with IL-1*β* enhanced the therapeutic efficacy of MSCs not only in intestinal IR injury but also in other acute organ damage by activating cellular COX-2-PGE_2_ signaling.

The third important observation of our study was that we used mRNA sequencing to explore the potential mechanisms by which activated ADMSCs might enhance healing in intestinal IR injury in a mouse model. In this study, we found that the mRNA expression profile was profoundly different between the groups treated with activated ADMSCs and the IR group. GO analysis of differentially expressed mRNAs revealed that processes falling in the categories of neutrophil chemotaxis, inflammatory response, and chemokine activity were involved in the healing of intestinal IR injury in the group treated with activated ADMSCs. Inhibition of neutrophil recruitment has been shown to be an important way for MSCs to ameliorate hepatic IR injury [32], while repression of the inflammatory response has been shown to be critical for the ability of MSCs to treat sepsis-induced organ injury [7], in line with our findings. Furthermore, KEGG pathway analysis suggested that some pathways, including the NF-*κ*B-P65 pathway, MAPK-ERK pathway, PI3K-AKT pathway, and PPAR pathway, were possibly involved in intestinal IR injury repair treated by activated ADMSCs. Western blot analysis further confirmed the intestinal IR-induced activation of the NF-*κ*B, MAPK-ERK, and PI3K-AKT pathways, while activated ADMSC treatment significantly repressed the activation of these pathways. Li et al. reported that MSCs ameliorated acute lung injury by inhibiting the LPS-induced upregulation of TLR2 and TLR4 expressions and suppressing NF-*κ*B-P65 phosphorylation and inflammatory reaction [34]. Additionally, downregulating MAPK-ERK1/2 phosphorylation was a demonstrated mechanism for inducing anti-inflammatory activity in macrophages [35], which play an important role in healing of IR injury and sepsis [36–38]. Finally, inhibiting COX-2-PGE_2_ signaling in activated ADMSCs abrogated their effects, including suppression of the activation of the NF-*κ*B-P65, MAPK-ERK1/2, and PI3K-AKT pathways induced by the intestinal IR injury.

There were some limitations to this study that should be acknowledged. First, we hypothesized that IL-1*β* enhances the therapeutic effects of ADMSCs via paracrine activity rather than by affecting differentiation. Therefore, we did not detect the cell fates of transplanted ADMSCs because previous studies reported that ADMSCs efficiently differentiate into mesenchymal lineages rather than epithelial cells [39, 40]. Second, although the therapeutic effects of activated ADMSCs with IL-1*β* have been demonstrated in a mouse model, some issues remained to be addressed before this treatment can be clinically applied, including poor engraftment and the potential tumourigenesis of transplanted ADMSCs [41] These problems should be fully evaluated in future studies.

## 5. Conclusion

In summary, the results presented in this study revealed that IL-1*β* activated ADMSCs via COX-2-PGE_2_ signaling in vitro and enhances the therapeutic effects of ADMSCs on intestinal IR injury by suppressing inflammation and apoptosis. In addition, we found that treatment with activated ADMSCs with IL-1*β* led to profound alterations in mRNA expression in intestinal IR injury and revealed that some biological processes, including neutrophil chemotaxis, inflammatory response, and chemokine activity, were involved. The NF-*κ*B-P65, MAPK-ERK1/2, and PI3K-AKT pathways activated by IR injury were suppressed by activated ADMSC treatment in intestinal IR-injured tissues. When COX-2-PGE_2_ signaling was inhibited in ADMSCs, the therapeutic effects of activated ADMSCs were impaired (Figure 8). Further work should focus on the clinical application of ADMSCs and explore dosing, timing, and suitable delivery approaches for this treatment in intestinal IR injury.

## Data Availability

The data used to support the findings of this study are available from the corresponding author upon request.

## Supplementary Materials

Supplementary Materials Supplementary Figure 1: identification of human ADMSCs. (A) ADMSCs displayed a spindle shape. (B, C) ADMSCs could be induced to differentiate into adipocytes and osteoblasts. (D–F) Flow cytometry showed that ADMSCs highly expressed CD29 and CD90, while lowly expressed CD45. Supplementary Figure 2: IL-1*β* increased expression of COX-2 and *α*-SMA at a dose- and time-dependent manner (A, B). Supplementary Figure 3: (A, B) IL-1*β* stimulated secretion of PGE2 from ADMSCs at a dose- and time-dependent manner. (C, D) Pretreatment with 50 ng/ml IL-1*β* did not induce differential secretion of IL-10 and TGF-*β*1 in ADMSCs (NS no statistical significance, Student's *t* test). Supplementary Figure 4: (A, B) Western blot and real-time RT-PCR were used to assess the effect of lentiviral shRNA for silencing of COX-2 expression in ADMSCs. (C, D) Inhibition of COX-2 using NS298 (COX-2 inhibitor) suppressed IL-1*β*-induced expression of COX-2 and *α*-SMA in ADMSCs. Supplementary Table 1: primer sequences for real-time RT-PCR (5′->3′).
