# Supplementary material for: Enhanced Effect of IL-1β-Activated Adipose-Derived MSCs (ADMSCs) on Repair of Intestinal Ischemia-Reperfusion Injury via COX-2-PGE2 Signaling
Source: Stem Cells Int. 2020 Apr 17;2020:2803747. doi: 10.1155/2020/2803747 (PMC7183531; doi:10.1155/2020/2803747)
Supplement: Supplementary Materials — Supplementary Figure 1: identification of human ADMSCs. (A) ADMSCs displayed a spindle shape. (B, C) ADMSCs could be induced to differentiate into adipocytes and osteoblasts. (D–F) Flow cytometry showed that ADMSCs highly expressed CD29 and CD90, while lowly expressed CD45. Supplementary Figure 2: IL-1β increased expression of COX-2 and α-SMA at a dose- and time-dependent manner (A, B). Supplementary Figure 3: (A, B) IL-1β stimulated secretion of PGE2 from ADMSCs at a dose- and time-dependent manner. (C, D) Pretreatment with 50 ng/ml IL-1β did not induce differential secretion of IL-10 and TGF-β1 in ADMSCs (NS no statistical significance, Student's t test). Supplementary Figure 4: (A, B) Western blot and real-time RT-PCR were used to assess the effect of lentiviral shRNA for silencing of COX-2 expression in ADMSCs. (C, D) Inhibition of COX-2 using NS298 (COX-2 inhibitor) suppressed IL-1β-induced expression of COX-2 and α-SMA in ADMSCs. Supplementary Table 1: primer sequences for real-time RT-PCR (5′->3′). [file 2803747.f1.pdf]

Supplementary figures

Supplementary figure 1 Identification of human ADMSCs. (**A**) ADMSCs displayed a spindle shape; (**B-C**) ADMSCs could be induced to differentiate into adipocytes and osteoblasts; (**D-F**) Flow cytometry showed that ADMSCs highly expressed CD29 and CD90, while lowly expressed CD45.

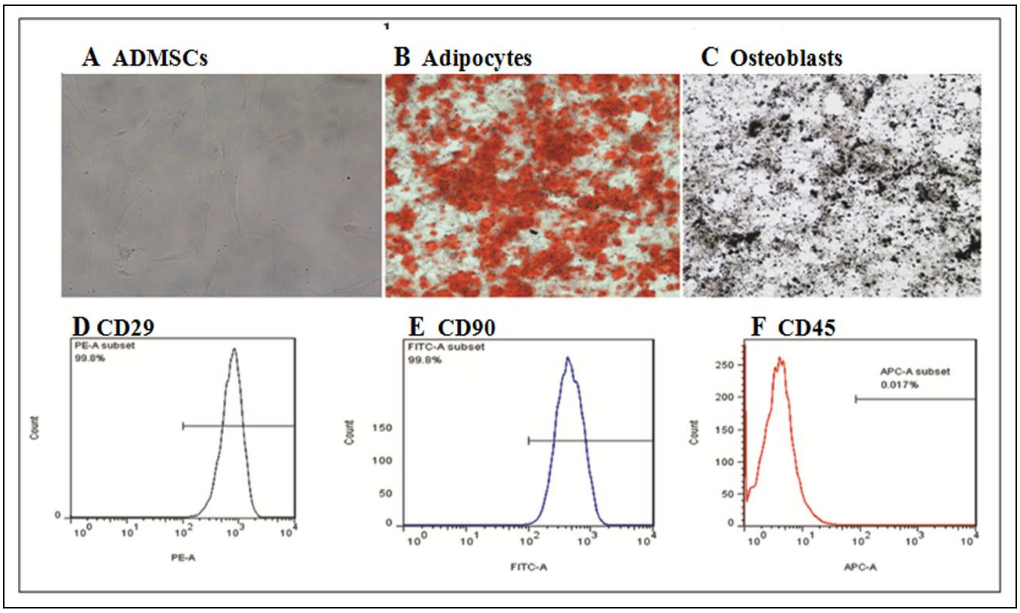

Supplementary figure 2 IL-1β increased expression of COX-2 and α-SMA at a dose- and time-dependent manner (A-B).

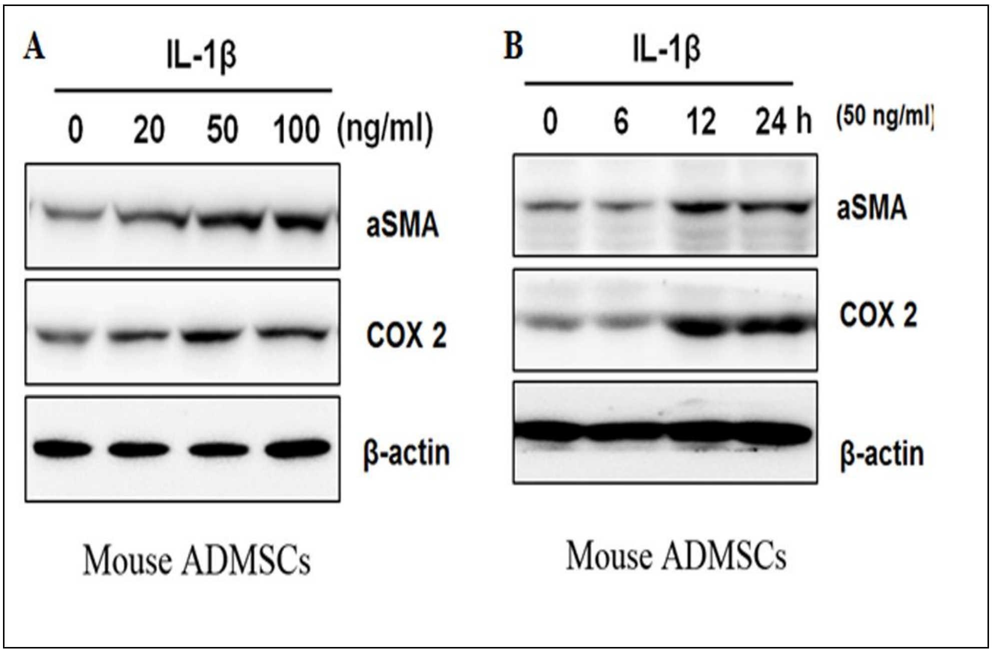

Supplementary figure 3 (**A-B**) IL-1β stimulated secretion of PGE_2 from ADMSCs at a dose- and time-dependent manner. (**C-D**) Pretreatment with 50ng/ml IL-1β did not induce differential secretion of IL-10 and TGF-β1 in ADMSCs (NS no statistical significance, student's t test). **(E-F)** IL-1β treatment induced ADMSCs senescence, including decreased cell proliferation, decreased expressions of p21 and p25 and cellular migration.

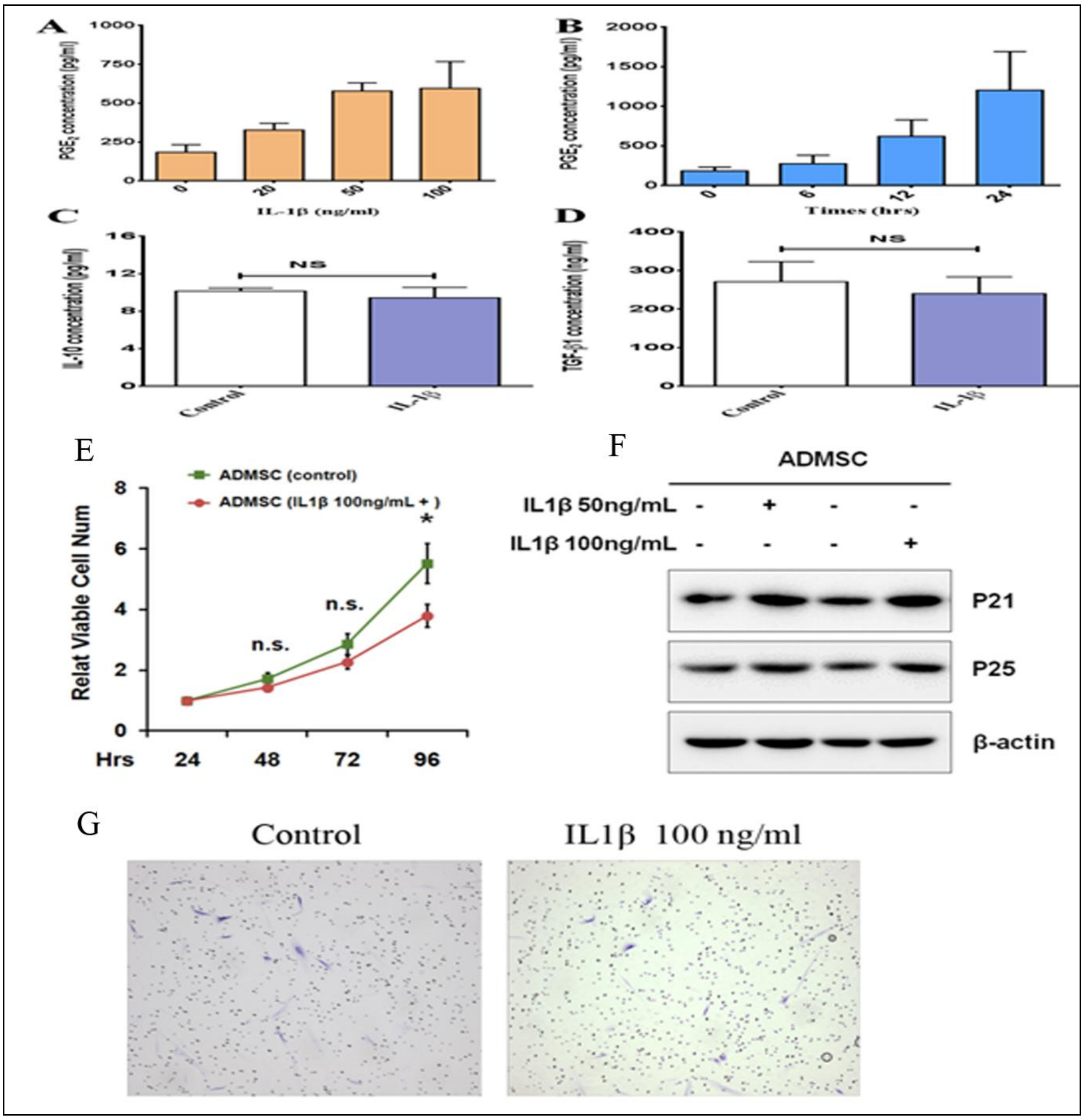

Supplementary figure 4 (**A-B**) Western blot and real-time RT-PCR were used to assess the effect of lentiviral shRNA for silencing of COX-2 expression in ADMSCs. (**C-D**) Inhibition of COX-2 using NS298 (COX-2 inhibitor) suppressed IL-1β-induced expression of COX-2 and α-SMA in ADMSCs.

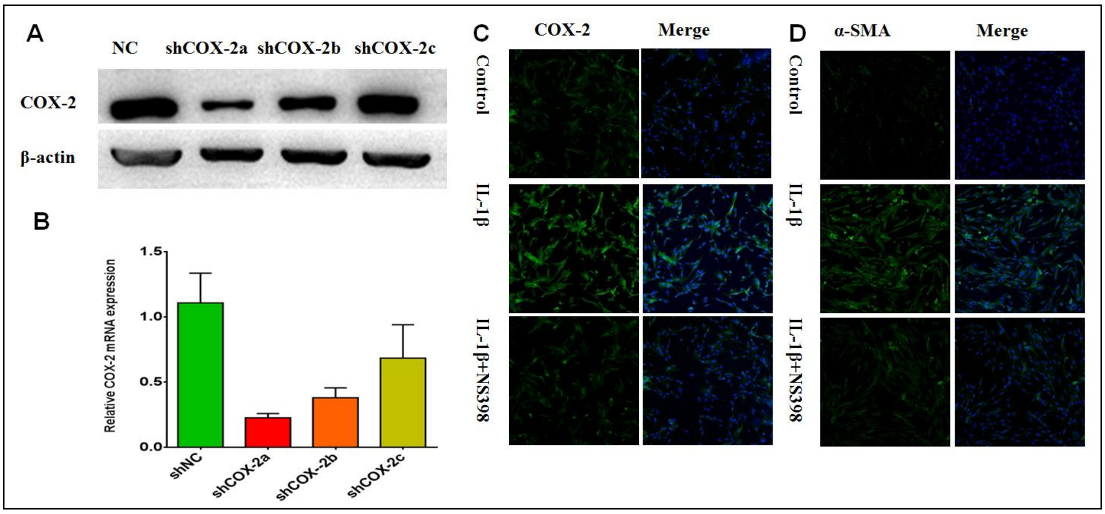

A
NC
shCOX-2a
shCOX-2b
shCOX-2c
COX-2
β-actin
B
Relative COX-2 mRNA expression
1.5
1.0
0.5
0.0
shNC
shCOX-2a
shCOX-2b
shCOX-2c
C
COX-2
Merge
Control
IL-1β
IL-1β+NS398
D
α-SMA
Merge
Control
IL-1β
IL-1β+NS398

Supplementary table 1 Primer sequences for real-time RT-PCR（5'-> 3'）

Gene name	Forward	Reverse
IL-6	CCTCTGGTCTTCTGGAGTACC	ACTCCTTCTGTGACTCCAGC
TNF-α	ATGAGCACAGAAAGCATGA	AGTAGACAGAAGAGCGTGGT
COX-2	GAAACTCTGGCTAGACAGCGTAA	AACCGTAGATGCTCAGGGAC
Apaf1	CAGTAATGGCGTCTTGTCAGT	AAGCGGCTGCTCGTTGATATT
S100a9	GCACAGTTGGCAACCTTTATG	TGATTGTCCTGGTTTGTGTCC
Tnfrsf22	AAATGTCCCGCTGGTGAATAC	GGCGGCACGATTCTGGAAA
cxcl2	CCAACCACCAGGCTACAGG	GCGTCACACTCAAGCTCTG
cxcl11	TGTAATTTACCCGAGTAACGGC	CACCTTTGTCGTTTATGAGCCTT
cxcr2	ATGCCCTCTATTCTGCCAGAT	GTGCTCCGGTTGTATAAGATGAC
cxcr4	GACTGGCATAGTCGGCAATG	AGAAGGGGAGTGTGATGACAAA
β-actin	GGCACCACACCTTCTACAATG	GGGGTGTTGAAGGTCTCAAAC
